# Data on the effect of lead concomitant noise on oxidative stress in rats

**DOI:** 10.1016/j.dib.2018.03.093

**Published:** 2018-03-26

**Authors:** Batol Masruri, Azadeh Ashtarinezhad, Parvaneh Yekzamani

**Affiliations:** a Department of Occupational Health, School of Public Health, Iran University of Medical Sciences, Tehran, Iran; b Student Research Committee, Faculty of Public Health, Iran University of Medical Sciences, Tehran, Iran

**Keywords:** Lipid peroxidation, Malondialdehyde, Oxidative stress, Lead acetate, Noise, Male rat

## Abstract

Exposure to chemical and physical factors occur in many occupations. Exposure to ambient pollutants such as noise, heavy metals, drugs enhance free radicals and can cause oxidative stress. The aim of the present project was to investigate noise and lead as two workplace stressors in rats. 20 male rats were assigned into 4 groups randomly. Rats in control group was not exposed to any stressor agent, while the first group was exposed to noise (105 dB, 4 kHz), the second group was exposed to lead acetate (gavage,4 mg/kg), and the last group was exposed to both lead and noise. In order to assess oxidative stress, the serum levels of malondialdehyde (MDA), as a product of lipid peroxidation was measured by thiobarbituric acid and also total antioxidant capacity (TAC) were measured by using ELISA kits. Our research showed significant enhancement in levels of malondialdehyde in exposed groups compare to control group. Also our results showed considerable decrease in levels of TAC in exposed groups compared to control group. Lead and noise exposure for 30 days caused a statistically significant enhancement in MDA level and significant decrease in the serum TAC level. On the other hand, statistically no significant difference was observed between the MDA and TAC levels between exposed groups. Moreover, body weight between exposed groups have decreased compared to control group. The outcomes of this study confirms the effect of noise and lead on lipid peroxidation. However, further studies are needed to clarify the mechanisms of oxidative stress through lead and noise exposure.

**Specifications Table**

**Table:** 

Subject area	Occupational health
More specific subject area	Biochemistry, Toxicology
Type of data	Table, figure
How data was acquired	All samples analyzed according to Aust and Buege method for MDA and TAC assay kit for TAC. Body weight also was measured with laboratorial scale.
Data format	Raw, analyzed
Experimental factors	Blood samples collected before and after exposure. MDA levels was measured by UV–vis spectrophotometer at 535 nm and TAC was determined at *λ* = 490 nm in a microplate reader.
Experimental features	The serum levels of MDA and TAC were determined.
Data source location	Tehran, Iran
Data accessibility	Data are reported in this article.

**Value of date:**

• The result of this study is useful for industrial workers that are exposed to lead concomitant noise as an environmental stressor
• The cumulative effect of noise plus lead as sub-acute exposure is the innovation of this work
• These data showed lipid peroxidation enhancement as a result of workplace stressor and can be helpful for many organizations such as ministry of labor and Ministry of Health and Medical Education.

## 1 Data

**Fig. 1:**
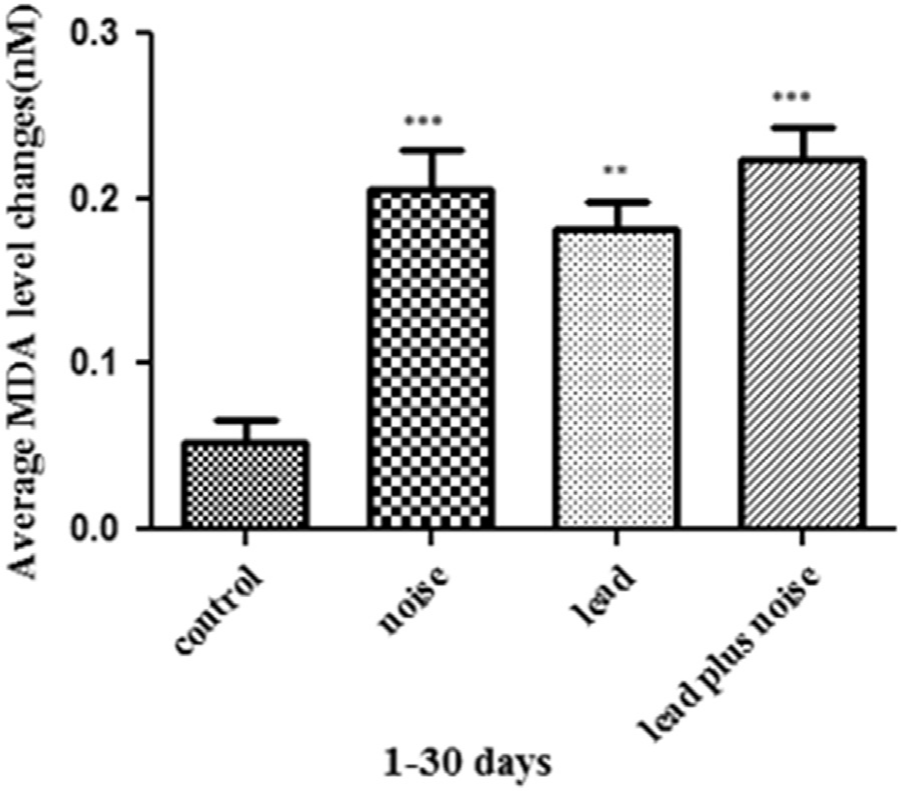
The effect of lead and noise on lipid peroxidation levels (Mean ± SD) between control and exposed groups (*n* = 5 rats) for 30 days. **P < 0.01 and ***P < 0.001 compared to control group.

MDA level in control and exposure groups are shown in Table 1. Even though our investigation suggests that there is significantly alteration in lipid peroxidation levels between control and exposed groups but statistically considerable relation was not observed among exposed groups. However, lipid peroxidation levels in lead plus noise group was different with both lead group and noise group. However, the difference was not statistically significant (Fig. 1).

**Table 1:** The serum MDA level (Mean ± SD) before and after exposure (*n* = 5 rats).

Groups	MDA (Before of exposure) nmol /g Hb	MDA) After 30 days (nmol /g Hb
Control	**0.21 ± 0.03**	**0.26 ± 0.04**
Noise	**0.19 ± 0.016**	**0.4 ± 0.03**
Lead	**0.21 ± 0.021**	**0.39 ± 0.015**
Lead plus noise	**0.2 ± 0.02**	**0.43 ± 0.05**

^*^P < 0.05 compared to control group.

**Fig. 2:**
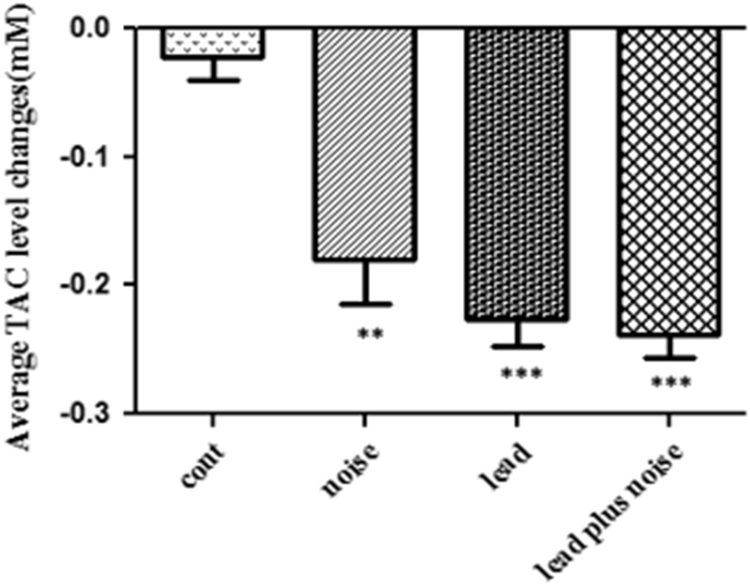
The effect of lead and noise on total antioxidant capacity levels (Mean ± SD) between control and exposed groups (*n* = 5 rats) for 30 days. **P < 0.01 and ***P < 0.001 compared to control group.

TAC level in exposed and control groups are shown in Table 2. As demonstrated in Fig. 2, TAC levels reduced significantly between exposed and control groups; however, the difference was negligible. Difference between body weight in various groups are also reported in Table 3. Based on our findings, there was no significant reduction in body weight among noise group and control group. The greatest reduction of body weight was observed between lead and lead plus noise groups compared to control group.

**Table 2:** The serum TAC level (Mean ± SD) before and after exposure (*n* = 5 rats).

Groups	TAC (Before of exposure) nmol/L	TAC)After 30 days ( nmol/L
Control	**0.15 ± 0.06**	**0.13 ± 0.76**
Noise	**0.21 ± 0.05**	**0.03 ± 0.042**
Lead	**0.25 ± 0.04**	**0.026 ± 0.025**
Lead plus noise	**0.24 ± 0.04**	**0.01 ± 0.065**

^*^P < 0.05 compared to control group.

**Table 3:** Body weight (Mean ± SD) in experimental groups (*n* = 5 rats).

Groups	Days		
	**0 day**	**15**th **day**	**30**th **day**
Control	**291.96 ± 5.99**	**305.35 ± 5.92**	**342.09 ± 5.97**
Noise	**293.77 ± 4.62**	**304.47 ± 4.66**	**335.62 ± 4.72**
Lead	**295.43 ± 422**	**282.6 ± 4.24**	**264.07 ± 4.14**
Lead plus noise	**298.95 ± 3.61**	**275.78 ± 4.97**	**256.55 ± 5.36**

^*^P < 0.05 compared to control group.

## 2 Experimental design, materials and method

### 2.1 Animal

20 healthy male rats weighting 250–300 g were utilized for this project and purchased from Experimental and Comparative Studies Center, Iran University of Medical Science, Tehran, Iran. They were housed under standard condition laboratory (12 h dark/ 12 h light cycle, 23 ± 2 °C temperature) [1].

The rats were fed with a standard diet and water freely [2], [3]. The Animal Ethics Committee of Iran University of Medical Science has verification of the experimental protocol.

### 2.2 Exposure

The rats were randomly divided into 4 groups: the first group was control, the second group exposed to noise; the third group was exposed to lead acetate by gavage and the last group was exposed to noise and lead acetate. Animals in control group received 1 ml/ day distilled water by gavage for 30 days. Animals in the second group were exposed to 4 kHz octave band at 105 dBA for 8 h/ day (occupational exposure) for 30 days [4], [5] and chamber was designed so that 10 rat heads can be placed there together. Third group received lead acetate by gavage for 30 days (4 mg/kg, lead acetate from MERCK and solution ready with distilled water) [6], and the last group exposed to noise and lead for 30 days. During the exposure with noise we measured noise intensity was measured via 4 point inside the chamber. Blood samples collected before (the first day) and after (the 30th day) exposure.

### 2.3 Measuring MDA level in serum

Lipid peroxidation is one of the reactions that increases the production of free radicals. Due to paroxysm of free radicals to lipids, are produced various aldehyde such as malondialdehyde that is one of the most common biomarkers of lipid peroxidation. MDA level enhancement indicated an abnormality in antioxidant defense mechanisms. In this study, MDA level in serum was measured according to Aust and Buege method [7], [8]. In this method, one molecule of MDA reacts with two molecules of thiobarbituric acid (TBARS) in acidic PH and high temperature and produces pink color. Based on this procedure, at first TCA–TBA–HCL solution were prepared include trichloro acetic acid %15 (w/v), thiobarbituric acid %0.375 (w/v) and Hydrochloric acid 0.25 N. Then, 1 ml of sample and 2 ml TCA–TBA–HCL solution were added and, finally samples were placed into hot water bath in 95 °C for 15 min. After chilling, they were centrifuged at 2000 rpm for 10 min and absorbance of upper layer was measured in UV–vis spectrophotometer at 535 nm against distilled water as blank. Concentration of MDA calculated by using Extinction coefficient 1.56*10^5^ M^-1^ cm^-1^ [9], [10]. The results were reported as nmol/g Hb.

### 2.4 Measuring TAC level in serum

The serum total antioxidant capacity was assayed using a colorimetric assay kit (Total antioxidant capacity assay kit, Zellbio GmbH Germany) according to the manufacturer's protocol. The optical density was determined at *λ* = 490 nm in a micro plate reader (Japan, BIORAD 680).

### 2.5 Statistical analysis

The data were analyzed with SPSS 22.0. All data were expressed as mean ± SD and then evaluated by using one-way analysis of variance (ANOVA) followed by Bonferroni procedure for multiple comparisons. *P* value was considered ≤ 0.05.
